# Bowel conservation in a case of giant jejuno-ileal duplication

**DOI:** 10.4103/0971-9261.71754

**Published:** 2010

**Authors:** Minakshi Sham, Dileep Phadke, Dasmit Singh

**Affiliations:** Department of Paediatric Surgery, B.J. Medical College & Sassoon General Hospitals, Pune - 411 001, India

**Keywords:** Bianchi technique, bowel conservation, giant jejuno-ileal duplications

## Abstract

The management of very long tubular bowel duplications poses a special challenge to even the most skilled surgeon. In these cases, mucosal stripping is usually employed. We report a novel case of a two-year-old boy, with 120 cm long jejuno-ileal duplication, wherein, bowel salvage was achieved, utilizing the Bianchi principle, originally described for bowel lengthening in cases of short bowel syndrome.

## INTRODUCTION

Duplications of the alimentary tract are rare. The treatment needs to be tailored for each case because of a wide spectrum of presentations. The salvage of almost 120 cms of small bowel by dissection through one of the mesentric leaves based on the Bianchi technique,[1,2] initially used for bowel lengthening in cases of short gut syndrome is reported.

## CASE REPORT

A two-year-old male child presented with abdominal pain of 15 days duration. There was no history of vomiting or constipation. He had a slightly distended abdomen. A vaguely palpable lump could be felt on abdominal examination. It occupied almost the entire abdomen. The child weighed 10 kg. His haematologic investigations were normal.

**Figure 1:**
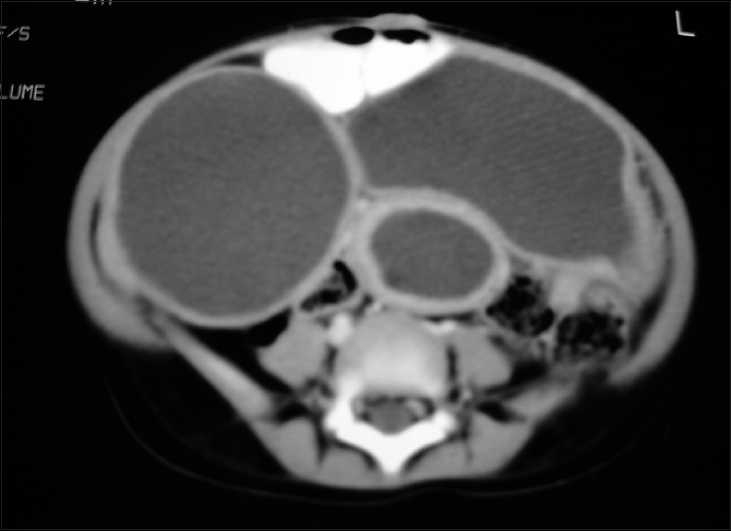
CT scan of the abdomen showing well-defined duplication cysts in the upper abdomen

An erect radiograph of the abdomen showed a well-defined, huge, soft tissue lesion in upper abdomen. There was paucity of gas in the abdomen and pelvis. Two to three different cysts were noted within the abdomen on ultrasonography. Computed tomogram (CT) scan of the abdomen showed a 10×5 cm large, cystic, lesion in the lower abdomen, continuous with the bowel wall [Figure 1].

**Figure 2:**
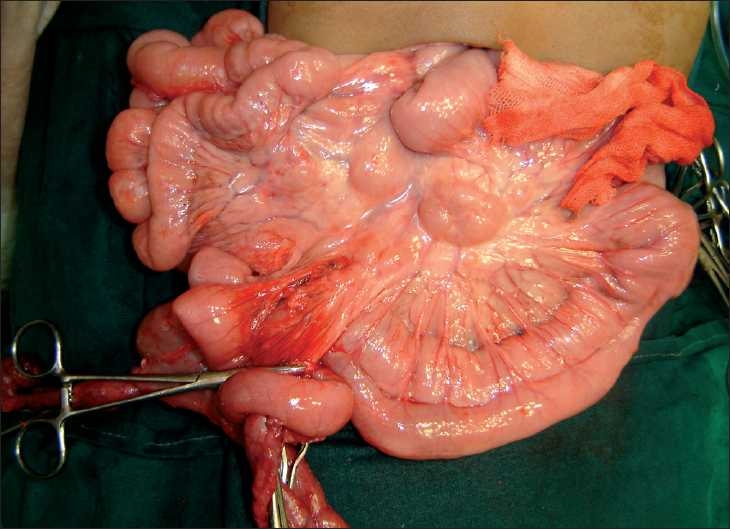
Very long tubular duplication cyst with the stretched out overlying jejunal segment

At laparotomy, cystic duplication of the jejunum was seen. A cyst measuring 12×10 cm in size was connected by a small stalk to a larger cyst of about 28×12 cm in size. There was no communication with the native jejunum. The blood vessels of the involved bowel passed both anterior and posterior to the cyst. Enucleation of the cysts was done by opening one leaf of the jejunal mesentery and preserving the vascularity of the bowel, through the posterior arcade. After near complete enucleation of the cystic duplication, another 70 cm long tubular duplication was found in continuity [Figure 2], invading up to the proximal ileum. Thus, only 30 cm of the terminal ileum and initial 20–25 cm of the proximal jejunum were uninvolved. This segment of duplication, however, shared the muscularis with the native small bowel. By gradual dissection through the anterior mesenteric leaf and division of the common muscularis between the native bowel and the tubular duplication, the involved jejunoileal segment of bowel could also be dissected free.

The tubular duplication was entering the terminal ileum at its distal end. There were a lot of hypertrophic mucosal folds at the entry point into the ileum. This portion (about 8 cm of bowel) was excised and local end-to-end anastomosis was done. The operative time was five hours. Blood loss of 60 cc was replaced. The dissection was aided by use of bipolar diathermy and operating loupes with 2.5×magnification.

The excised mass weighed 750 g and contained viscid mucous. The histopathology showed the intestinal type lining mucosa. The patient had an uneventful post-operative recovery. At 1-year follow-up, the child is healthy and asymptomatic.

## DISCUSSION

**Figure 3:**
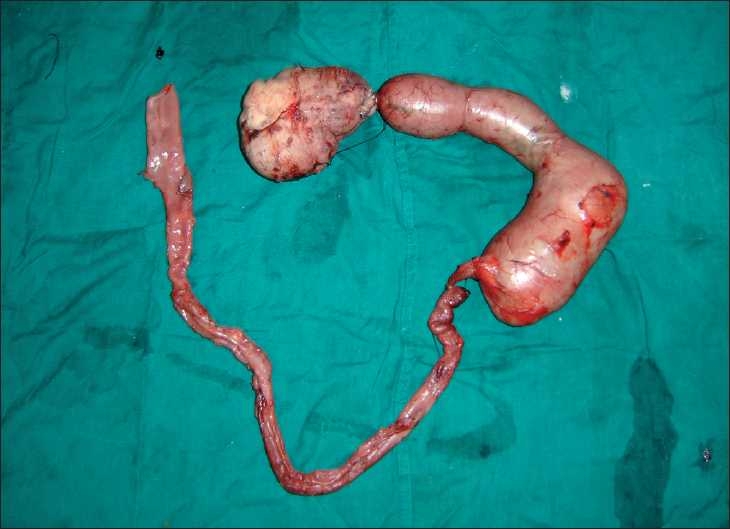
The duplication specimens placed in continuity as per their order of presentation during the surgery

In our case, there were two large cystic duplications i.e., 12×10 cm and 28×12 cm of the proximal jejunum, connected to each other by a small stalk. This was further continuous with a 70 cm long tubular duplication [Figure 3]. The cystic duplications had a distinctly separate wall, whereas, the tubular duplication shared a common muscularis with the native bowel.

In this situation, excision of the involved bowel would have significantly compromised the bowel length. Hence, we enucleated the two large cystic duplications and separated the long tubular duplication from the native bowel by dissection of the common wall. By coagulating and dividing the overlying blood vessels of the anterior mesenteric leaf based on Bianchi principle,[1] salvage of entire small bowel except 8-10 cm of distalmost portion of the tubular duplication was possible.

In the Bianchi technique, the entire circumference of the bowel is divided into two halves, each of which is supplied by blood vessel from one mesenteric leaf.[3] Whereas, in our case, since the duplication shared the blood supply with the native bowel, quite a few blood vessels from the anterior leaf of mesentery required cauterization for excision of the giant jejuno-ileal duplication and yet, the vascularity of the bowel was well maintained, predominantly by the blood vessels from the posterior arcade.

Though such type of dissection has been utilized for limited lengths of small bowel,[3] preservation of blood supply in spite of dissection for almost 120 cm in a two-year old child has not been described so far.

High degree of technical expertise, patient bowel handling, gentle dissection using bipolar diathermy and magnifying operating loupes and vigilant post-op care salvaged the small bowel in this two-year old child.
